# Moment Feature Based Fast Feature Extraction Algorithm for Moving Object Detection Using Aerial Images

**DOI:** 10.1371/journal.pone.0126212

**Published:** 2015-06-01

**Authors:** A. F. M. Saifuddin Saif, Anton Satria Prabuwono, Zainal Rasyid Mahayuddin

**Affiliations:** 1 Faculty of Information Science and Technology, University Kebangsaan Malaysia, 43600 UKM Bangi, Selangor D.E., Malaysia; 2 Faculty of Computing and Information Technology, King Abdulaziz University, P.O. Box 344, Rabigh, 21911, Saudi Arabia; Imperial College London, UNITED KINGDOM

## Abstract

Fast and computationally less complex feature extraction for moving object detection using aerial images from unmanned aerial vehicles (UAVs) remains as an elusive goal in the field of computer vision research. The types of features used in current studies concerningmoving object detection are typically chosen based on improving detection rate rather than on providing fast and computationally less complex feature extraction methods. Because moving object detection using aerial images from UAVs involves motion as seen from a certain altitude, effective and fast feature extraction is a vital issue for optimum detection performance. This research proposes a two-layer bucket approach based on a new feature extraction algorithm referred to as the moment-based feature extraction algorithm (MFEA). Because a moment represents thecoherent intensity of pixels and motion estimation is a motion pixel intensity measurement, this research used this relation to develop the proposed algorithm. The experimental results reveal the successful performance of the proposed MFEA algorithm and the proposed methodology.

## Introduction

The significance of feature extraction using aerial images from unmanned aerial vehicles (UAVs) has increased in the field of computer vision with the development of moving object detection algorithms using aerial images. The purpose of efficient feature extraction is to facilitate fast moving object extraction using aerial images from UAVs in the frame achieved via two-frame difference methods. Appropriate feature selection is a challenging task due to the large number of features that can be extracted, which requires a substantial amount of processing time during the detection process. In addition, certainimage types, such as aerial images, must be scanned at multiple orientations and scales with hundreds of thousands of windows. This paper presents a two-layer bucket (TLB) approach based on a new feature extraction algorithm named the moment-based feature extraction algorithm (MFEA), which is expected to bridge the gap between fast and less complex feature extraction algorithms for moving object detection using aerial images from UAVs.

The computation time and complexity of detection typically depend on the types of features used. In previous research, three types of features have been used for moving object detection, i.e., corner [1–4], color [2, 4–6], and edge [1, 4, 7, 8] features. The most recently obtained detection speed using the corner feature was 6.25 fps [9], and 6 fps was obtained using the color feature [10]. In addition, edge feature detection is capable of achieving 24.2 fps [11]. For corner-based moving object detection, the Harris corner is the most commonly used technique. For edge detection, several types of edge detectors have been used, e.g.,Sobel, Canny, and Prewitt [1, 12, 13]. Recently, numerousresearchers have started to use corner and edge features together [1, 4, 14–16]. However, almost all of the previous researchers did not attempt to attain decreased computation times either using color, corner, or edge features separately or using an integrated process. This research proposes a new feature extraction algorithm named the MFEA,according to which momentsare extracted as features from aerial images.

## Background

Previous motion-based moving object detection methods require various parameter estimation techniques using differenttypes of features. Substantial parameter estimationprocesses currently requirelargecomputation timesgiven thecomputation complexity demands for new feature extraction algorithmsbecause aerial images must be captured from different altitudes.

The significance of feature extraction using aerial images has increased with the development of aerial image-based moving object detection in the computer vision research field. The purpose of efficient feature extraction is to facilitate fast moving object extraction from aerial images in a given frame based on frame difference methods. Appropriate feature selection is a challenging task due to the large number of features present in a typical frame,requiringa significant amount of processing time during the detection process. Moreover, nearly all of the previous research was concentrated only on detection rate rather than reducing the computational complexity while maintaininghigh detection rate. Because motion detection and the detection of a moving object are coupled, a less complex feature extraction algorithm is needed to ensure proper motion estimation and the detection of objects with less computation time and lower computational complexity.

Typically, computation time and the complexity of detection performance depend on the type of feature used. In previous research, three types of features were used for moving object detection, i.e., corner [1–4], color [4–6], and edge [7, 8] features. The most recent detection speedachieved using corner features was 6.25 fps [17]; for color features,6 fps [6]; and for edge features, the detection speed was 24.2 fps [18]. For corner-based moving object detection, the Harris corner is the most commonly used technique. For edge detection, there are several types of detectors,e.g., Sobel, Canny, and Prewitt [1, 12, 13]. Recently, many researchers have started using corner and edge features together [4, 14, 15, 18]. However, almost all of the previous researchers did not attempt to decrease the computation time, either by using color, corner, and edge features separately or by an integrated process.

### Color Feature

The work in [1] used color features via extendingpixel-wise classification method by preserving relations among neighboring pixels in a region. Due to its dependence on large parameter estimations, the proposed research did not provide sufficient reliability. The work in [19] used color features by identifying candidate key points of object pixels. Due to the dependency on the structural shape, theproposed research did not perform well. The work in [6] used color features for complex backgrounds inurban environments. Given the constraint of using grayscale input images, real time detection [6] cannot be considered as a reliable solution.

### Corner Feature

The research presented in [2] used corner features by implementing a motion analysis methodin which motion was achieved by using the frame difference method. However, their research concentrated only on the detection rate,and no evaluation was performed to measure the computational complexity for achieving a computation time measurement. Only one dataset PVD was used in [2], for which the detection rate was merely 50%. The work in [10] used corner features. Larger feature sets were extracted from neighboring pixels, and a dual selection approach was used to reduce the computation complexity of feature selection. Their proposed method did not provide the expected results for unstructured objects, the presence of stark contrasts, the presence of long shadows, the reflection of sunlight, rectangular triangular structures on the tops of buildings, and objects in parking spots when the objects weresituated in parallel. The work in [20] used corner features to overcome challenges of the system by consistently addressing 3D image orientation, image blurring due to airplane vibrations, variations in illumination conditions, and season changes. However, their proposed method rejects most the object background for their input aerial images, which is unrealistic. The researchers in [21, 22] used corner features by implementing a context-aware saliency detection algorithm associated with the surrounded environment to segment points that attract attention in human vision. Although their research did not provide sufficient experimental evidence, their work provides good results in terms of shape resolution and the variant appearance of object, which overcomes the short-comings of traditional segmentation algorithms and is suitable for aerial image segmentation.

### Edge Feature

The work in [14] used edge features,wherein the researchers proposed a new feature extraction framework using shadows in conjunction with the rotationally invariant shape matching of edge features using shape context descriptors extracted from object edges. Due to the dependency on lightening conditions, the work in [14] cannot identify objects for clocked shadows. The researchers in [15] used edge features for images that exhibit low quality and pose variations across the set as a result of changes in object location and articulation. Their proposed method exhibited better performance and increased persistence in high-frame-rate videos because the method obeys the assumption that the object position in the next frame should be close to its position in the current frame. The researchers in [4] used edge features by clustering single points obtained from motion estimations. Their research did not provide the expected results in terms of the complexity of shortening environment, real-time changes in background, and inconspicuous features of objects. In [13, 18], theresearchers used edge features in individual frames in terms of data association, which was highly challenging and ambiguous. Because their proposed research must be sufficiently discriminative for data association to be performed across long periods of partial and full occlusions, their research results wereunreliable due to substantial dependencies on a classifier, which increased thecomputer complexity. The researchers in [23, 24] used edge features based on motion compensation and analysis. However, their proposed research did not overcome traditional problems of motion-analysis-based moving object detection depending on a substantial number of parameters.

After performing a comprehensive review, we note that none of the previous research approaches used moment features for moving object detection using aerial images from UAVs. In addition, almost all of the previous research focused on improving detection rate rather than reducing computational complexity while maintaining a high detection rate. Because motion detection is coupled with the detection of objects, a less complex feature extraction algorithm must bedeveloped to ensure proper motion estimation and object detection with minimal computation time and complexity. In other words, motion estimation indicates the detection of motion pixels, the performance of which can be described as a function of the image pixel intensity as well as pixel color value. With regard to images, a moment in computer vision and probability theory also carries the same meaning in image features for detecting moving object using aerial images from UAVs.

This research proposes the use of image moment features for moving object detection using aerial images from UAVs and presents a new feature extraction algorithm referred to as the MFEA, which exhibits a reduced computational time and is less complex compared with algorithms that use other features.

## Proposed Research Methodology

The proposed moment-based feature extraction framework is depicted in the proposed framework section, and the two-layer bucket framework is depicted in theTLB section, where a new algorithm named the MFEA is proposed. Each section of the methodology is proposed with a new approach to ensure the robustness and accuracy of the detection methodology.

### Proposed Framework

In the proposed framework, a TLBapproach, which acts as temporary storage space of moment-based motion features and is used to reduce computational complexity and decrease computation time, was adopted. Given that frame differences alone can obtain only single-pixel point motion instead of complete object motion and that segmentation does not have the ability to differentiate moving regions from the basic static region background, this research used segmentation and frame difference together to achieve optimum detection performance for moving object detection using aerial images from UAVs. The proposed frameworkis presentedin S1 Fig.

If F_A_(x,y,t) and F_A_(x,y,t-1) are two consecutive frames corresponding to consecutive times t and (t-1), then the frame difference F_f_(x,y,t) is defined by Eq (1).

$${\text{F}}_{\text{f}}\text{(x, y, t) = round}\left({\text{F}}_{\text{A}}{\text{(x, y, t)-F}}_{\text{A}}\text{(x, y, t-1)}\right)$$ (1)

F_f_(x,y,t) can be defined using Eq (2).

$$\left\{\begin{array}{cc} {\text{F}}_{\text{f}}{\text{(x, y, t) = F}}_{\text{B}}\text{(x, y, t)} & {\text{if(F}}_{\text{f}}\text{ > 0)}\\ {\text{F}}_{\text{f}}{\text{(x, y, t) = F}}_{\text{A}}\text{(x, y, t)} & {\text{if(F}}_{\text{f}}\text{ < 0)} \end{array}\right\}$$ (2)

### Moment-based Matrix Formation

Let I_f_(x,y,t) be the median filtered result from F_f_(x,y,t). If x and y are the co-ordinatesof I_f_(x,y,t), the raw moments of I_f_(x,y) for order (p + q) can be defined as Eq (3).

$${\text{M}}_{\text{pq}}\text{ = }{\sum_{\text{p}}\sum_{\text{q}}\text{x}}^{\text{p}}{\text{y}}^{\text{q}}{\text{I}}_{\text{f}}\text{(x, y)}$$ (3)

When considering I_f_(x,y) as a 2D continuous function, Eq (3) can be expressed as

$${\text{M}}_{\text{pq}}\text{ = }\iint {\text{x}}^{\text{p}}{\text{y}}^{\text{q}}{\text{I}}_{\text{f}}\text{(x, y),}$$ (4)

Where theCentroidcoordinates are as follow:

$$\bar{\text{x}}\text{ = }\frac{{\text{M}}_{\text{10}}}{{\text{M}}_{\text{00}}}\text{ And y = }\frac{{\text{M}}_{\text{01}}}{{\text{M}}_{\text{00}}}\;{\text{; M}}_{\text{00}}\text{ = Zeroth moment = }\sum_{\text{m}}\sum_{\text{n}}{\text{m}}^{\text{0}}{\text{n}}^{\text{0}}\text{FD (m, n) = }\sum_{\text{m}}\sum_{\text{n}}\text{FD (m, n)}$$

Let I_p_(x,y) be obtained using the pixel intensity distribution for every pixel, which can be calculated using Eq (4) based on the pixel format of I_f_(x,y) for the co-ordinate (m,n), as shown in S2 Fig.

### Two-layer Bucket

Let M_T_ denote the total moment, Feature_T_ denote the total number of features, and w and H denote the width and height of I_p_(x,y), respectively. Themoment weight factor (MWF) is defined by Eq (5).

$$\text{MWF = Math}{\text{.log(M}}_{\text{T}}{\text{*Feature}}_{\text{T}}\text{*W*H)}$$ (5)

Then, I_p_(x,y) is decomposed into I_h_(x,y) and I_1_(x,y) based on the MWF acquired from the resultant of the following condition.

$$\left\{\begin{array}{cc} {\text{I}}_{\text{h}}\text{(x, y)} & \text{MWF > }\left|{\text{I}}_{\text{p}}\text{(x, y))}\right|\\ {\text{I}}_{\text{l}}\text{(x, y)} & \text{MWF < }\left|{\text{I}}_{\text{p}}\text{(x, y)}\right| \end{array}\right\}\text{,}$$ (6)

Where I_h_(x,y) contains the high intensity of the moment and I_l_(x,y) contains the low intensity of the moment. The decomposition of I_p_(x,y) into I_h_(x,y) and I_1_(x,y) is referred to here as the TLB process. I_h_(x,y) and I_l_(x,y) are considered to be the temporary stack of moment features that precede the segmentation to extract moving object. This research employed segmentation using color-based edge differences for the extraction of moving objects. Color-difference-based edge segmentation for every(x,y)of I_p_(x,y) can be defined as presentedin Eq (7).

$$\left\{\begin{array}{cc} {\text{I}}_{\text{e}}\text{(x, y) = K(q, r) + L(i, j) + M(s, t)} & \text{if(x,y)}\in {\text{I}}_{\text{h}}\text{(x,y)}\\ {\text{I}}_{\text{e}}\text{(x,y) }\neq \text{ K(q, r) + L(i, j) + M(s, t)} & \text{if(x,y)}\in {\text{I}}_{\text{l}}\text{(x,y)} \end{array}\right\}$$ (7)

For two pixels (g,h) and (a,b), three combinations of RGB color differences, K(q,r),L(i,j),M(s,t), are defined in Eq (8).

$$\begin{array}{l} \text{K(q, r) = Math.Abs(b.GetPixel(a, b)).B-b.GetPixel(g, h).B)}\\ \text{L(i, j) = Math.Abs(b.GetPixel(a, b)).G-b.GetPixel(g, h).G)}\\ \text{M(s,t) = Math.Abs(b.GetPixel(a, b)).R-b.GetPixel(g, h).R)} \end{array}$$ (8)

This research presents a feature extraction algorithm referred to as the MFEA, which is presented in S3 Fig.

## Experiment and Discussion

This research used the C Sharp programming language for the experimental analysis. Because this work used aerial images, we developed a raw-coded frame extractor and denoise tools using a median filter for the experimental analysis. The experimental analysis demonstrated the performance of the proposed MFEA algorithm in terms of the detection rate in comparison with several state-of-art processes,i.e., [5] those using color features, [12, 15, 17] edge features, [2, 3] and corner features. In addition, various experiments wereperformed using Sobel, Prewitt, Canny edge-based detection and Harris corner-based moving object detection to compare the detection rate, computation time, and complexity with the proposed MFEA algorithm in the same dataset mentioned in the dataset section.

### Datasets

This research used two UAV video data sets (S1 and S2 Videos) from the Center for Research in Computer Vision (CRCV) at the University of Central Florida (www.crcv.edu/data/ucf_aerial_action.php). An RC-controlled blimp equipped with a HD camera was used to obtain these datasets. The collected data represent a diverse pool of action features at different heights and from different aerial viewpoints. Multiple instances of each action were recorded at different altitudes, which ranged from 400 to 500 feet and were performed with different actors.

### Result

This research extracted 395 frames using a frame rate of 1 frame/second from the S1 Video video datasets and 529 frames using the same frame rate from the S2 Video video data sets. The frame size is 355 X 216. This section presents the experimental analysis and the results for the proposed MFEA algorithm. To evaluate the MFEA algorithm, two metrics, the detection rate (DR) and the false alarm rate (FAR), are defined based on the parameters presented in S4 Fig.

Detailed measurements for the true positive (TP), false positive (FP), false negative (FN), detectionrate (DR), and false alarm rate (FAR)metrics areprovided in S1 Table. The detection rate for MFEA is 82.23% for dataset S2 Video whenusing edge features, whereas [17], [15], and [12] demonstrated detection rates of70, 66, and 56%, respectively, using corner features. In addition, [2] and [3] demonstrated detection rates of50 and 75%, respectively;and [5] demonstrated a detection rate ofapproximately 65% using only color features. The detection rates for MFEA with other state-of-art methods are presented in S5 Fig.

Here, dataset 1 and dataset 2 indicate S1 and S2 Videos, respectively, and N denotes the total number of frames extracted from each data set. The relation between the detection rate and the false alarm rate is presented in S6 Fig and indicates that the number of frames usedproportionally increases the detection rate. In addition, the use of an increased number of frames decreases the false alarm rate.

To ensure the same hardware performance evaluation, this research evaluated the proposed MFEA in terms of the Detection Rate (DR) on Action1.mpg for different kinds of features, such as anedge-, corner- and moment-based new feature extraction algorithm, orMFEA, using 1 frame per second. The proposed MFEA is compared with other edge feature detection algorithms using 1 fps, forwhich each frame of the MFEA achieved a higher detection rate. At 1 fps, MFFA achieved 75.16% while the Sobel, Prewitt and Canny edge-based detection approaches achieved detection rates of 60.45%, 60.08% and 60.23%, respectively, as shown in S7 Fig. The proposed MFEA is compared with corner feature-based moving detection algorithm, where the MFEA achieved a higher detection at 1 fps, as shown in S8 Fig At 1 fps, the Moravec, Susan and Harris corner-based detection rates are 63.31%, 62.62% and 62.90%, respectively, as shown in S8 Fig, where as the MFEA achieved a detection rate of 75.16%.

### Computation Time

To obtain a computation measurement andensure thesame hardware performance, theproposed MFEA was evaluated in terms of the Computation Time on Action1.mpg for different kinds of features, such as the edge-, corner- and finally moment-based new feature extraction algorithm MFEA at 1 frame per second. The computationtime is measured based on an edge-based feature extraction and a corner-based feature extraction technique and compared with the MFEA proposed in this research. The proposed MFEA required a computation time of 0.589s; in [21], the computation required 3.97s using corner featuresand in [8], the computation time was 0.92s using edge features, as shown in S9 Fig.

For the same data set mentioned above, the Prewitt edge-based detection method requires the least amount of time (0.651s),whereas the Canny edge technique requires 0.668s. The Sobel edge-based detection method requires the greatest amount of time (0.768s) as shown in S10 Fig.

For the corner-feature-based detection approach, only the Harris corner-based approach provides good results (0.668s), whereas theother two corner-based approaches, theMoravec and Susan corner-based detection approaches, require 0.702s and 0.82s, respectively, as shown in S11 Fig.

Among all these feature extraction methods, the MFEA requires the shortest computation time as shown in S9, S10 and S11 Figs All of the previous methods use 3x3 matrix multiplication along with image width and height convolution, whereas the proposed MFEA uses moment features based on aTLB approach, which reduces the computation time to 0.589s as shown in S9, S10 and S11 Figs. The proposed algorithm categorized 45,984 low-density features for the 101^st^ frame from a total of 518,400 pixels and thus ignores these 45,984 features during computation, which decreases the computation time and complexity. In contrast, the studies in [2, 5, 12, 15, 17] and other approaches, such as the Sobel, Canny, and Prewitt edge-based and Harris and Susan corner-based moving object detection, consider all of the feature positions during object extraction.

### Computational Complexity

The proposed algorithm exhibits less computational complexity compared with edge-based detection, i.e., Canny and Sobel, and corner-based detection, i.e., Harris, and Susan, for moving object detection using aerial images from UAVs.

Due to the convolution of the image with a kernel, the computation of the gradient direction, and non-maximum suppression, edge-based detections, such as Canny and Sobel edge-based detectionsystems, exhibitcomplexities of Log(N) and NxN, respectively, whereasHarris and Susan corner-based detection exhibit complexities of Log(NxN) as shown in S12 Fig.

Edge-based detection using Sobel and Cannydetection is presented in S13 and S14 Figs, respectively. Corner-based feature detection using Moravec, Susan, and Harris detection is shown in S15, S16 and S17 Figs, respectively. Moment-based moving object detection using MFEA is presented in S18 Fig.

This work measures DR and FAR based on the number of frames extracted from video dataset inputs. The studies in [2, 3, 12, 14, 15] used various features, such as colors, corners, and edges. This research proposed a new feature extraction algorithm, MFEA, which combines frame difference and segmentation approaches and achieved a detection rate of 82.23% (for the video data set S2 Video). This result is a good indication of the optimum performance of moving object detection using aerial images from UAVs. In addition, MFEAproduces good results and is a fast feature extraction algorithm given that itexhibits a lower computation time compared with theother methods mentionedabove, which are considered state-of-the-art methods.

## Conclusion

The main purpose of this research is to present a new feature extraction algorithm for a fast and computationally less complex feature extraction technique that ensures optimum detection performance for moving object detection using aerial images from UAVs. The newly proposed feature extraction algorithm, MFEA, is based on a TLBapproach using high-and low-intensity pixels with the moment-based pixel intensity probability distribution. This study determined moments for all neighboring pixels of each pixel, thereby ensuring that very few pixels are missing and leading to the faster extraction of potential moving objects based on the moment estimation. The proposed MFEA demonstrated a detection rate of 82.23%, which is higher than the rates obtained by previous state-of-the-art methods and false alarm rates of 19.78%, which is the lowest rate relative to other feature-based object detection approaches, i.e., edges and corners. Based on the experimental results, the proposed moment-based feature extraction technique exhibits a low computation time, which indicates low complexity when extracting moving objects using aerial images from UAVs compared with other types of feature-based methods, such as those using colors, corners, and edges. To ensure the same hardware detection performance, the proposed MFEA was evaluated in terms of the detection rate and computation time to measure its computational complexity.

## Supporting Information

S1 Fig Proposed framework for moment-based fast feature extraction. The proposed framework involves six main parts. The input image must be determined by the frame difference approach, in which denoise effects are applied. Then, the main contribution of this research, the Two Layer Bucket Approach, is applied. After Segmentation using Edge Based Dilation is applied, the Moving object is detected using threshold effects.
(TIF)

S2 Fig Pixel format of I_p_(x,y) for the co-ordinate(x,y). (TIF)

S3 Fig MFEA algorithm for moving object detection. The proposed MFEA feature extraction algorithm describes the overall detection procedure, forwhich theMoment Weight Factor (MWF) is defined using Eq 5. I_h_(x_i_,y_j_) and I_l_(x_i_,y_j_)represent the high-intensity array of pixels bucket and the low-intensity pixels bucket, respectively, and both are separated into the main edge bucket I_e_(m,n) = based on the MWF condition. Finally, the moving object is determined by I_e_(x_i_,y_j_).
(TIF)

S4 Fig Dependency used to evaluate the moment-based feature extraction algorithm (MFEA). Performance evaluation of the proposed methodology is performed based on the Detection Rate (DR) and the False Alarm Rate (FAR). Both metrics depend on a common parameter, named True Positive (TP), where False Negative (FN) is related to the Detection Rate and False Positive (FP) is related to the False Alarm Rate.
(TIF)

S5 Fig Detection rates for MFEA with other state-of-art methods using different features. The performance of MFEA is compared with the edge, corner and color feature-based extraction methods described in previous works. Using the edge features presented in [11, 16, 22] provided detection rates of 70%, 66% and 56%, respectively, whereas using the corner features [2] yielded a detection rate of 50%. In addition, using the color feature [6] provided a detection rate of 75%. The proposed MFEA demonstrated a detection rate of 82.23%.
(TIF)

S6 Fig Detection rate and false alarm rate using the MFEA for two data sets. Two data sets were used to evaluate the performance of the proposed MFEA. The total frames extracted from two data sets,S1 and S2 Videos, were 395 and 527, respectively, based on a speed of 1 frame per second. S2 Video exhibited the higher detection rate along with a lower false alarm rate compared with the S1 Video data set.
(TIF)

S7 Fig Detection rate among Sobel, Prewitt, Canny edge based detection and Proposed MFEA. To ensure the same hardware performance evaluation, the research presented evaluated the proposed MFEA in terms of the Detection Rate (DR) for, S1 Video Actions1.mpg and different kinds of edge based detection i.e. Sobel,Prewitt and Canny, with 1 frame per second where MFEA exhibited higher detection rate.
(TIF)

S8 Fig Detection rate among Moravec, Susan, Harris corner based detection and Proposed MFEA. Proposed MFEA is also evaluated in comparison with corner based detection i.e. Moravec, Susan and Harris corner based detection to ensure the same hardware performance evaluation where MFEA also exhibited higher detection rate.
(TIF)

S9 Fig Computation time for MFEA with other state-of-art methods using different features. MFEA computation time is compared with the corner and edge feature-based object detection approaches. MFEA provides the lowest computation time of 0.589s, whereas the previous works in [13] and [15] provide computation times of 3.97s and 0.92s for the corner and edge features, respectively.
(TIF)

S10 Fig Computation time among various edge-based feature methods and MFEA on the S1 Video dataset. MFEA computation time is compared with several edge feature-based object detection methods. MFEA provides the lowest computation time of 0.589s, whereas the Sobel, Prewitt and Canny edge-based detection methods provide computation times of 0.787s, 0.665s and 0.688s, respectively.
(TIF)

S11 Fig Computation time among the various corner-based features and MFEA on the S1 Video dataset. MFEA computation time is compared with several corner feature-based object detection methods. MFEA provides the lowest computation time of 0.589s, whereas the Moravec, Susan and Harris corner-based detection methods provide computation times of 0.702s, 0.82s and 0.887s, respectively.
(TIF)

S12 Fig Complexity of MFEA and other methods. Based on the computation time and matrix multiplication, the MFEA algorithm exhibits a computationally less complex feature extraction approach than other methods. The complexity of MFEA is N, whereas the Canny, Sobel, Harris, and Susan based feature extraction complexities are Log (N), NxN, Log (NxN) and Log (NxN), respectively.
(TIF)

S13 Fig Detection using Sobel edge features. (TIF)

S14 Fig Detection using Canny edge features. (TIF)

S15 Fig Detection using Susan corner features. (TIF)

S16 Fig Detection using Moravec corner features. (TIF)

S17 Fig Detection using Harris corner features. (TIF)

S18 Fig Detection using moment features with MFEA. (TIF)

S1 Video First Video Datasets. S1 Video is named as data set 1 collected from the Center for Research in Computer Vision (CRCV) at the University of Central Florida (www.crcv.edu/data/ucf_aerial_action.php). This research extracted 395 frames using a frame rate of 1 frame/second from the S1 Video video datasets.
(MP4)

S2 Video First Video Datasets. S2 Video is named as data set 2 collected from the Center for Research in Computer Vision (CRCV) at the University of Central Florida (www.crcv.edu/data/ucf_aerial_action.php). This research extracted 529 frames using a frame rate of 1 frame/second from the S2 Video video data sets.
(MPG)

S1 Table Measurements of true positive (TP), false positive (FP), false negative (FN), detection rate (DR), and false alarm rate (FAR). (TIF)
