# Supplementary figures and images for: Moment Feature Based Fast Feature Extraction Algorithm for Moving Object Detection Using Aerial Images
Source: PLoS One. 2015 Jun 1;10(6):e0126212. doi: 10.1371/journal.pone.0126212 (PMC4452346; doi:10.1371/journal.pone.0126212)

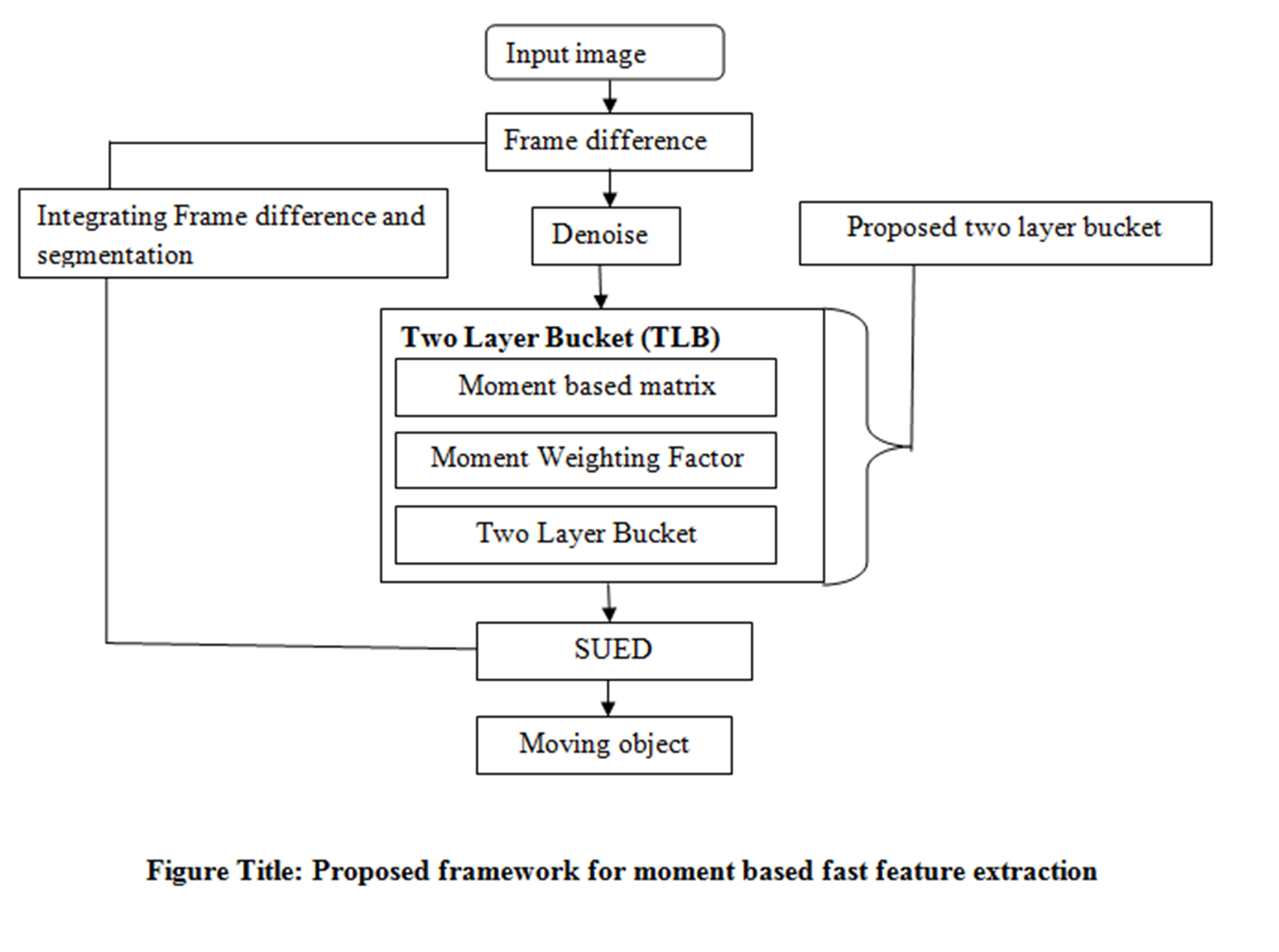

Supplement: S1 Fig — The proposed framework involves six main parts. The input image must be determined by the frame difference approach, in which denoise effects are applied. Then, the main contribution of this research, the Two Layer Bucket Approach, is applied. After Segmentation using Edge Based Dilation is applied, the Moving object is detected using threshold effects. (TIF) [file pone.0126212.s001.tif]

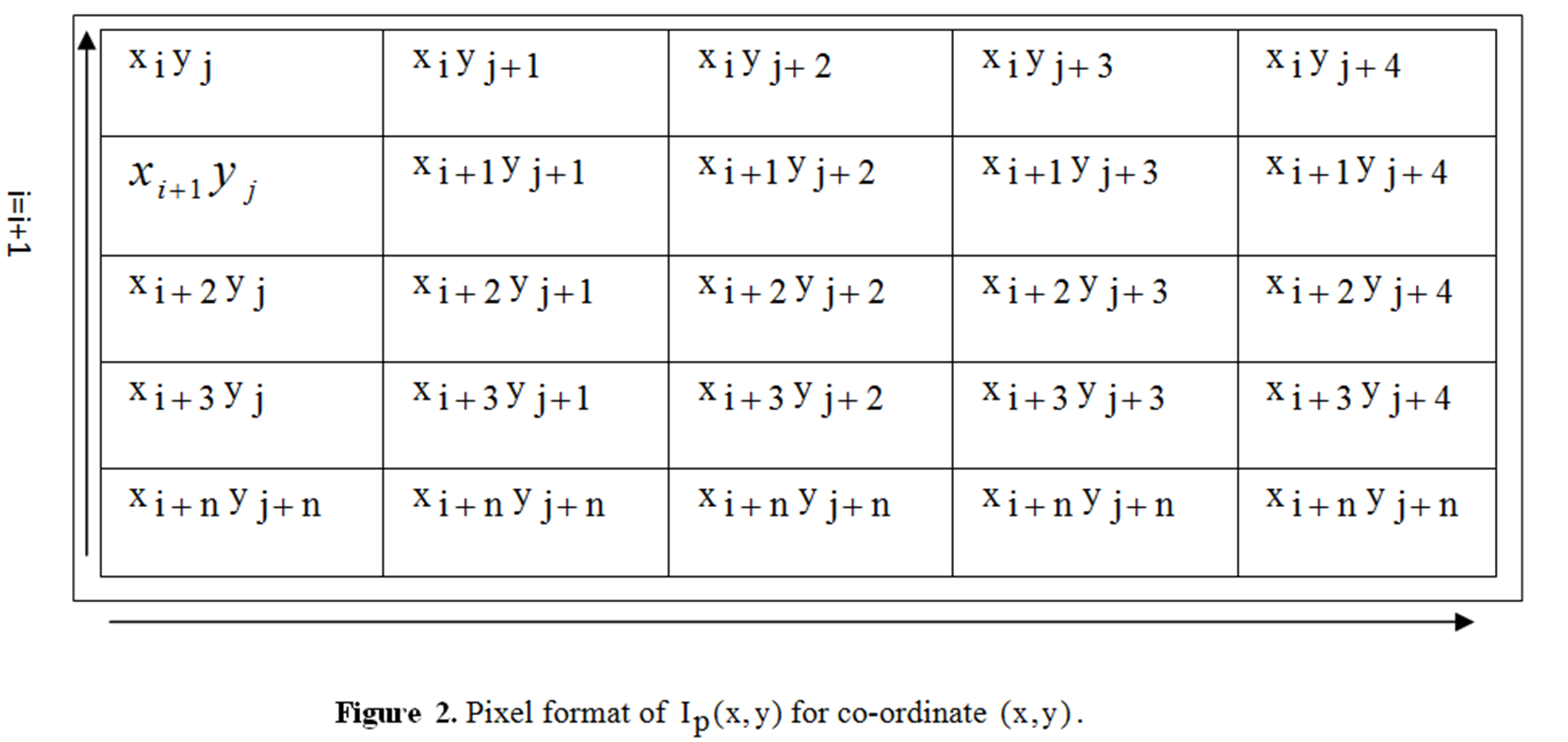

Supplement: S2 Fig — (TIF) [file pone.0126212.s002.tif]

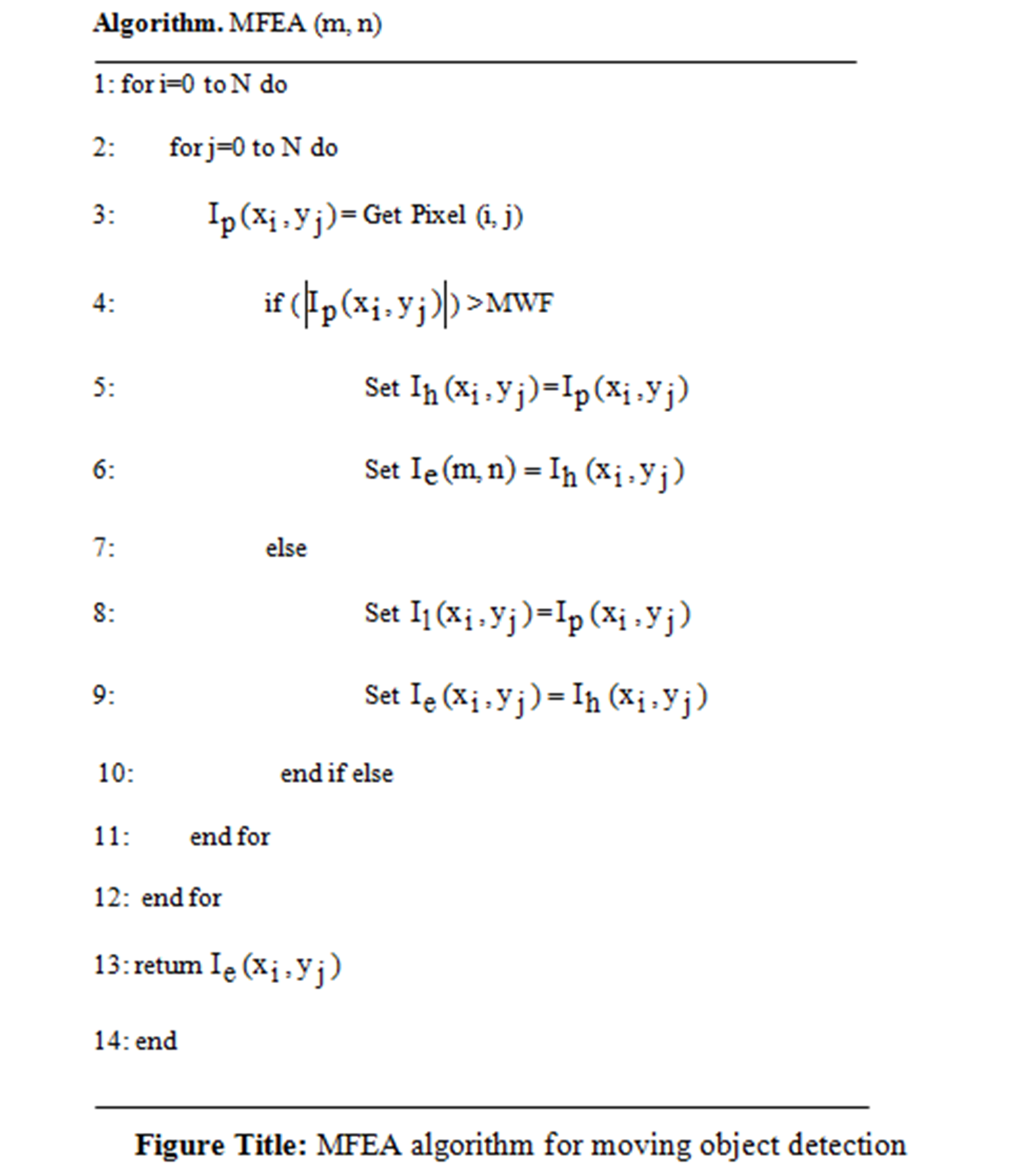

Supplement: S3 Fig — The proposed MFEA feature extraction algorithm describes the overall detection procedure, forwhich theMoment Weight Factor (MWF) is defined using Eq 5. Ih(xi,yj) and Il(xi,yj)represent the high-intensity array of pixels bucket and the low-intensity pixels bucket, respectively, and both are separated into the main edge bucket Ie(m,n) = based on the MWF condition. Finally, the moving object is determined by Ie(xi,yj). (TIF) [file pone.0126212.s003.tif]

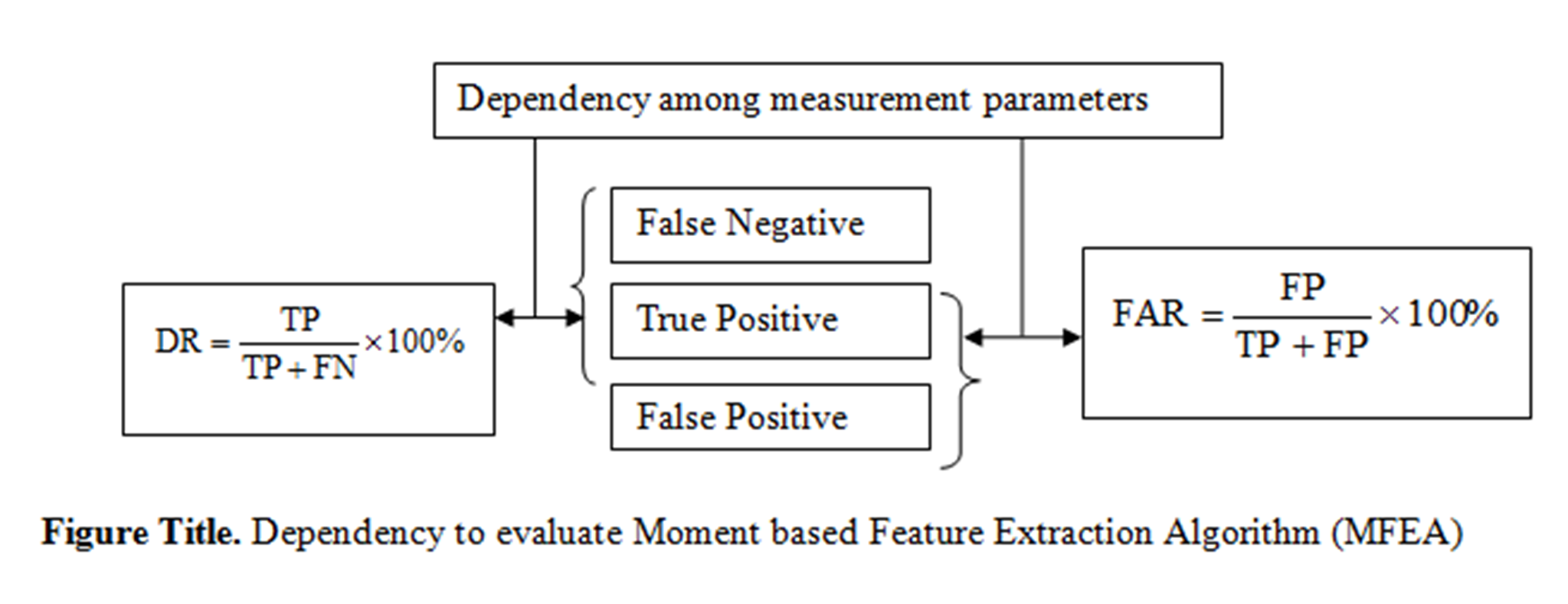

Supplement: S4 Fig — Performance evaluation of the proposed methodology is performed based on the Detection Rate (DR) and the False Alarm Rate (FAR). Both metrics depend on a common parameter, named True Positive (TP), where False Negative (FN) is related to the Detection Rate and False Positive (FP) is related to the False Alarm Rate. (TIF) [file pone.0126212.s004.tif]

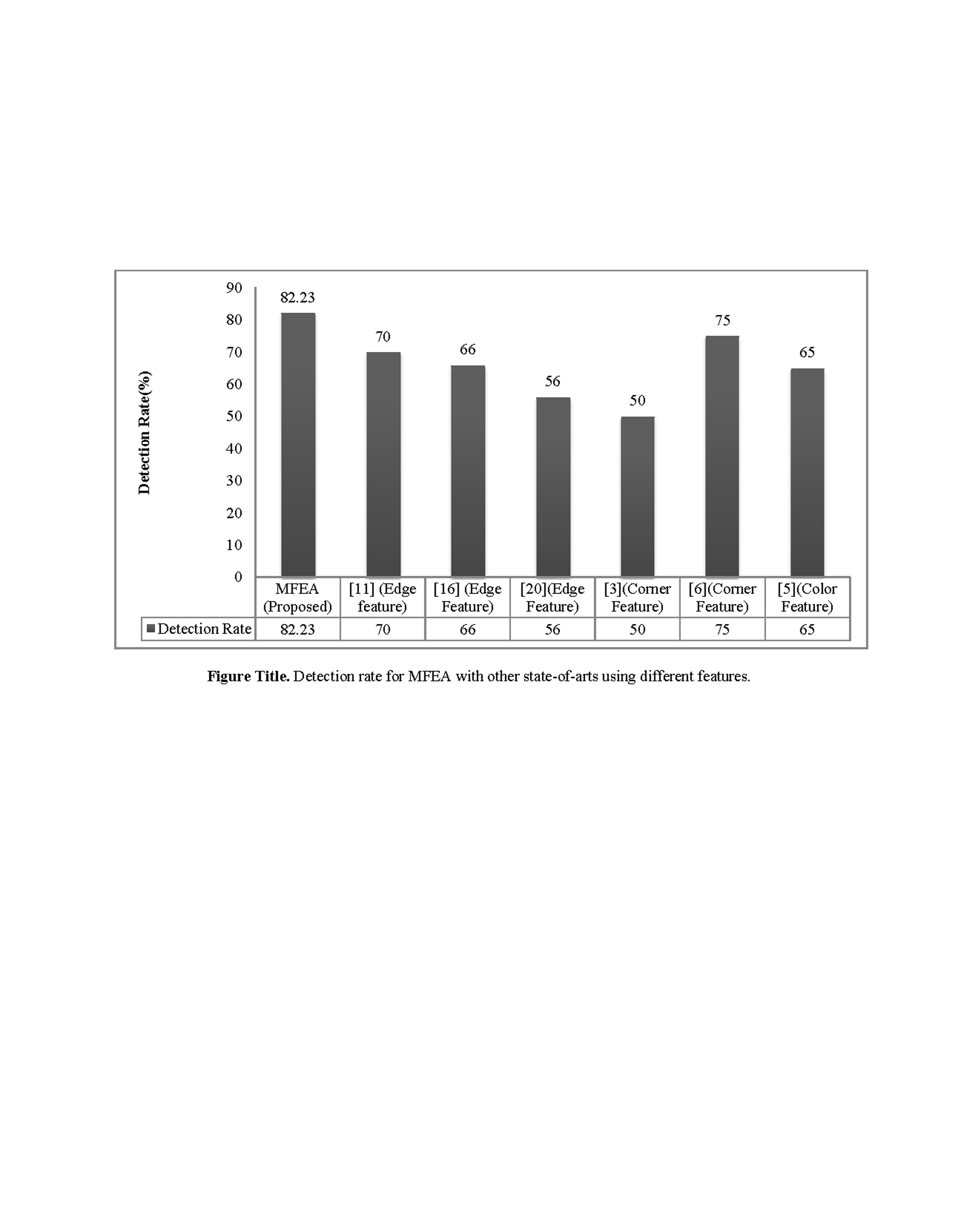

Supplement: S5 Fig — The performance of MFEA is compared with the edge, corner and color feature-based extraction methods described in previous works. Using the edge features presented in [11, 16, 22] provided detection rates of 70%, 66% and 56%, respectively, whereas using the corner features [2] yielded a detection rate of 50%. In addition, using the color feature [6] provided a detection rate of 75%. The proposed MFEA demonstrated a detection rate of 82.23%. (TIF) [file pone.0126212.s005.tif]

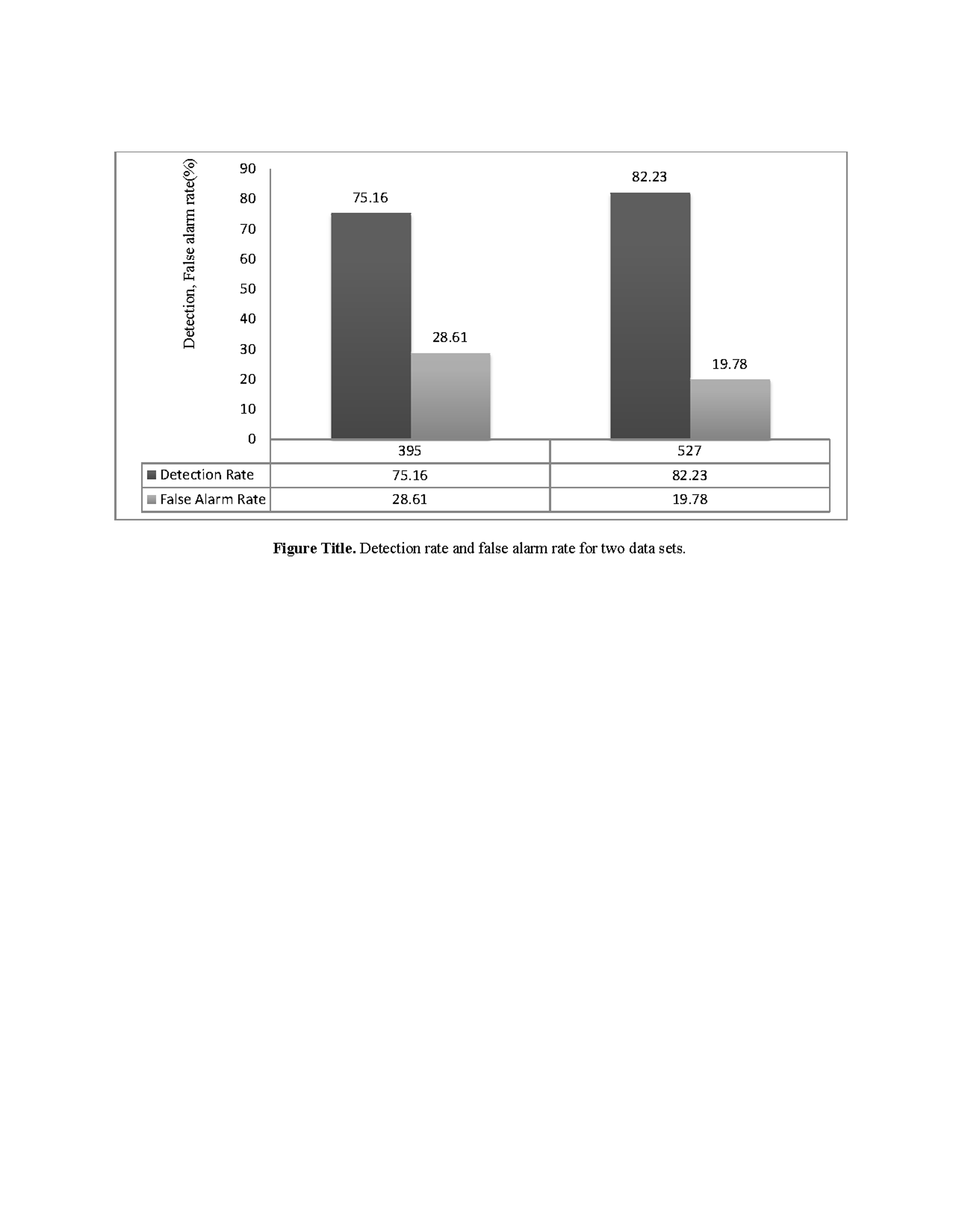

Supplement: S6 Fig — Two data sets were used to evaluate the performance of the proposed MFEA. The total frames extracted from two data sets,S1 and S2 Videos, were 395 and 527, respectively, based on a speed of 1 frame per second. S2 Video exhibited the higher detection rate along with a lower false alarm rate compared with the S1 Video data set. (TIF) [file pone.0126212.s006.tif]

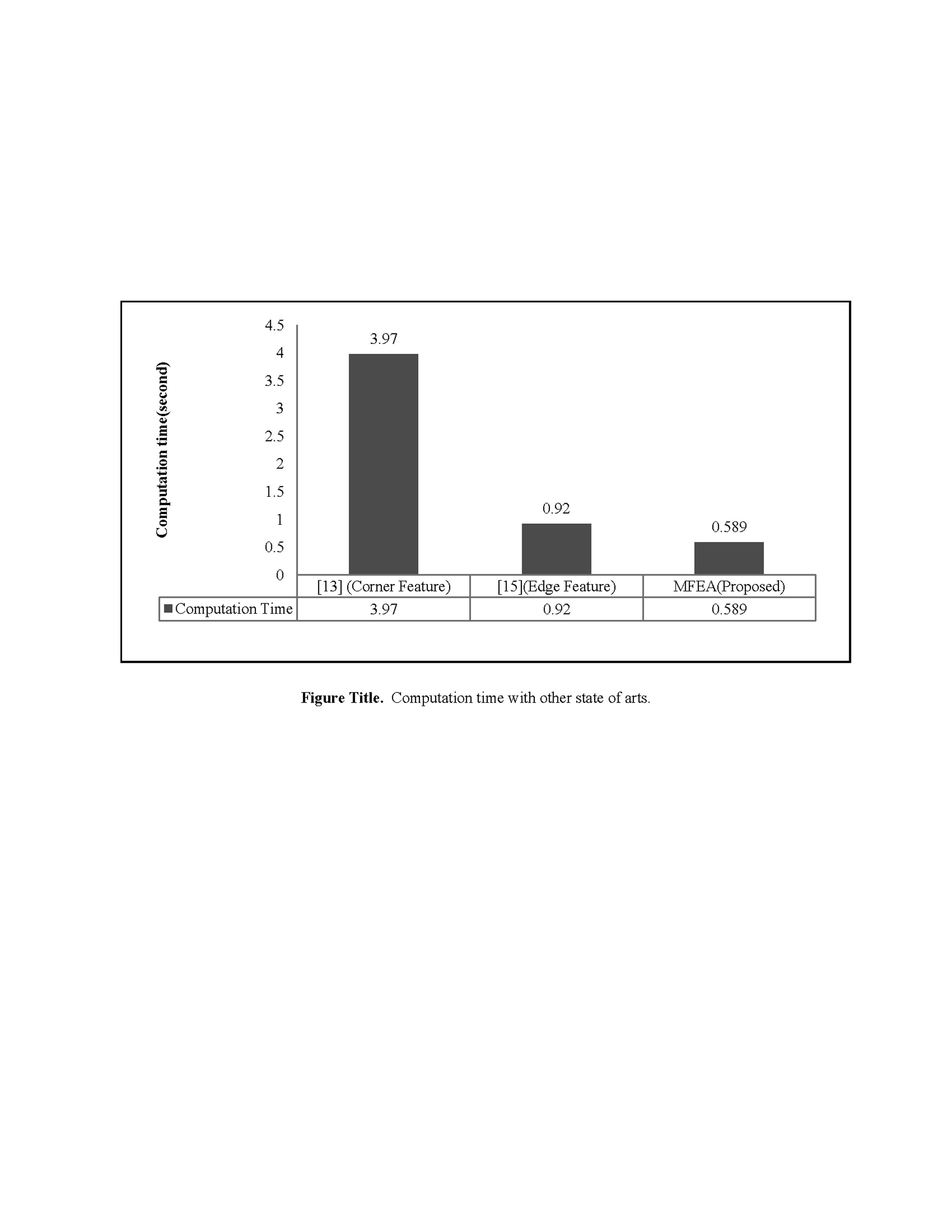

Supplement: S7 Fig — To ensure the same hardware performance evaluation, the research presented evaluated the proposed MFEA in terms of the Detection Rate (DR) for, S1 Video Actions1.mpg and different kinds of edge based detection i.e. Sobel,Prewitt and Canny, with 1 frame per second where MFEA exhibited higher detection rate. (TIF) [file pone.0126212.s007.tif]

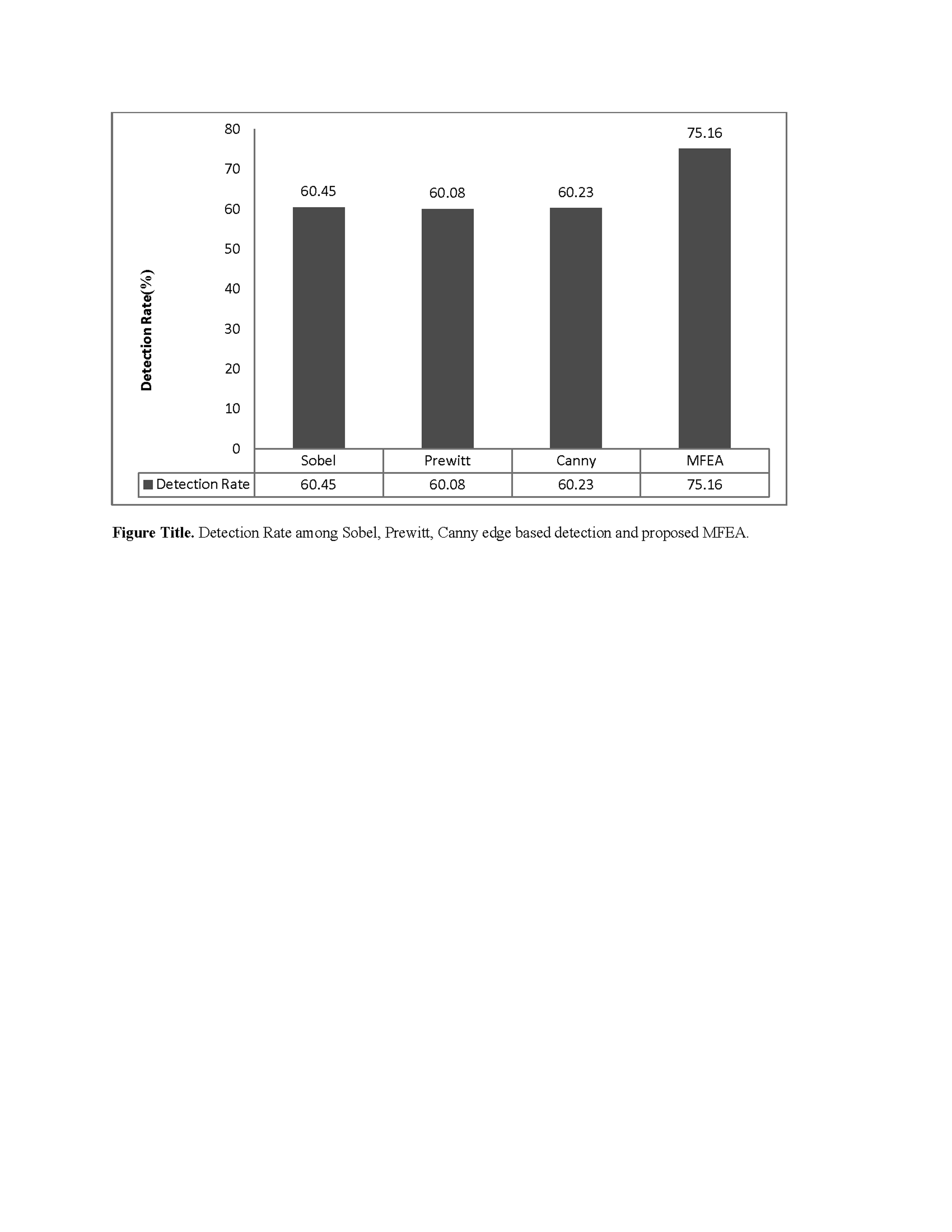

Supplement: S8 Fig — Proposed MFEA is also evaluated in comparison with corner based detection i.e. Moravec, Susan and Harris corner based detection to ensure the same hardware performance evaluation where MFEA also exhibited higher detection rate. (TIF) [file pone.0126212.s008.tif]

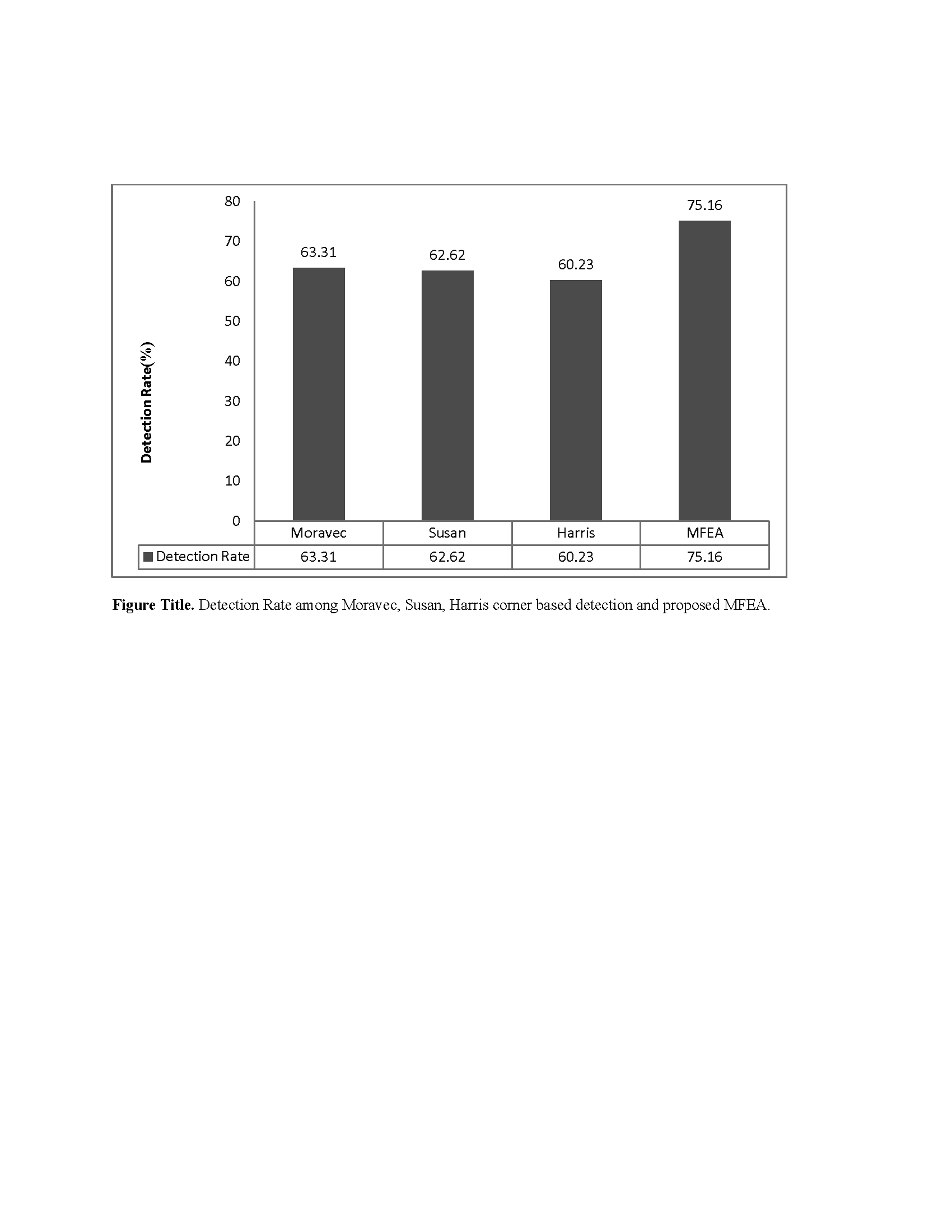

Supplement: S9 Fig — MFEA computation time is compared with the corner and edge feature-based object detection approaches. MFEA provides the lowest computation time of 0.589s, whereas the previous works in [13] and [15] provide computation times of 3.97s and 0.92s for the corner and edge features, respectively. (TIF) [file pone.0126212.s009.tif]

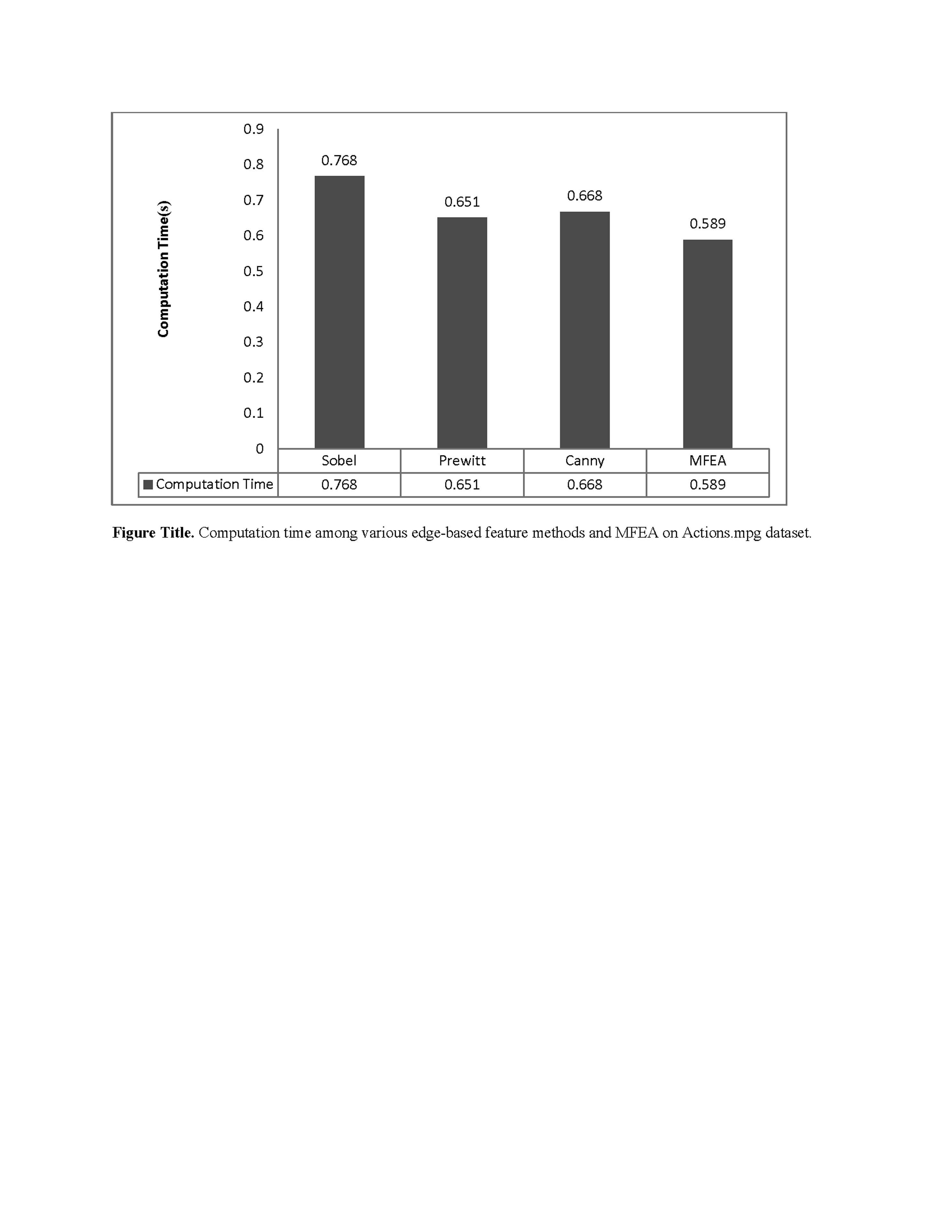

Supplement: S10 Fig — MFEA computation time is compared with several edge feature-based object detection methods. MFEA provides the lowest computation time of 0.589s, whereas the Sobel, Prewitt and Canny edge-based detection methods provide computation times of 0.787s, 0.665s and 0.688s, respectively. (TIF) [file pone.0126212.s010.tif]

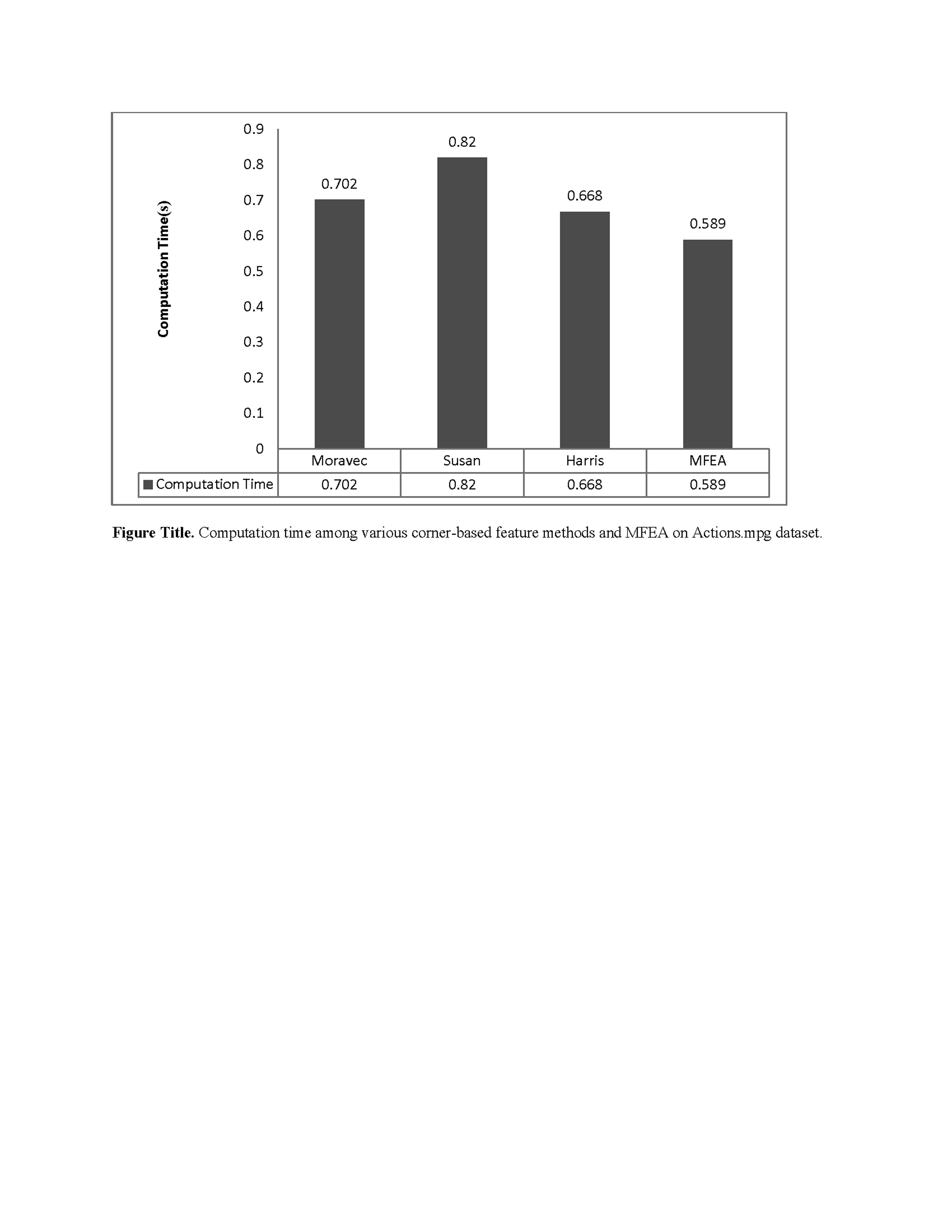

Supplement: S11 Fig — MFEA computation time is compared with several corner feature-based object detection methods. MFEA provides the lowest computation time of 0.589s, whereas the Moravec, Susan and Harris corner-based detection methods provide computation times of 0.702s, 0.82s and 0.887s, respectively. (TIF) [file pone.0126212.s011.tif]

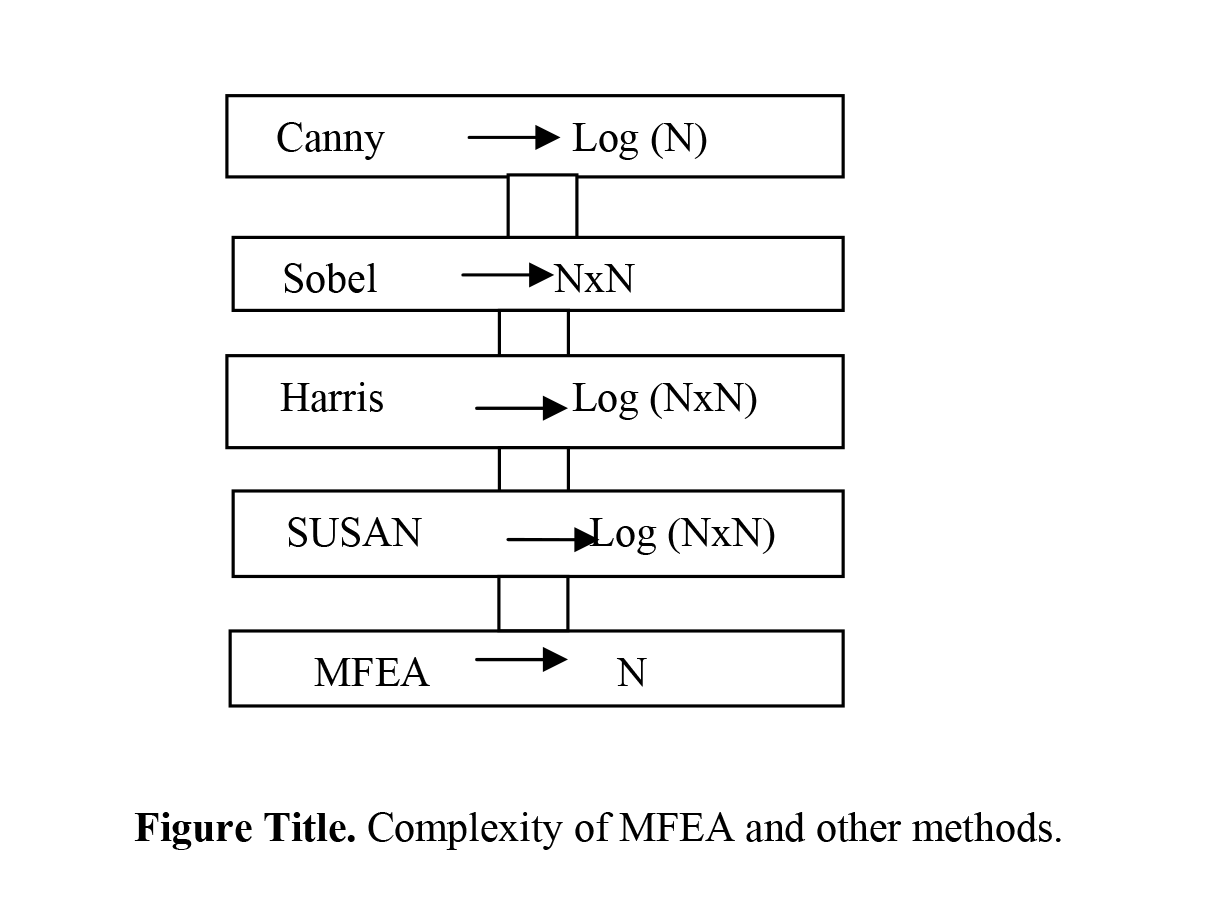

Supplement: S12 Fig — Based on the computation time and matrix multiplication, the MFEA algorithm exhibits a computationally less complex feature extraction approach than other methods. The complexity of MFEA is N, whereas the Canny, Sobel, Harris, and Susan based feature extraction complexities are Log (N), NxN, Log (NxN) and Log (NxN), respectively. (TIF) [file pone.0126212.s012.tif]

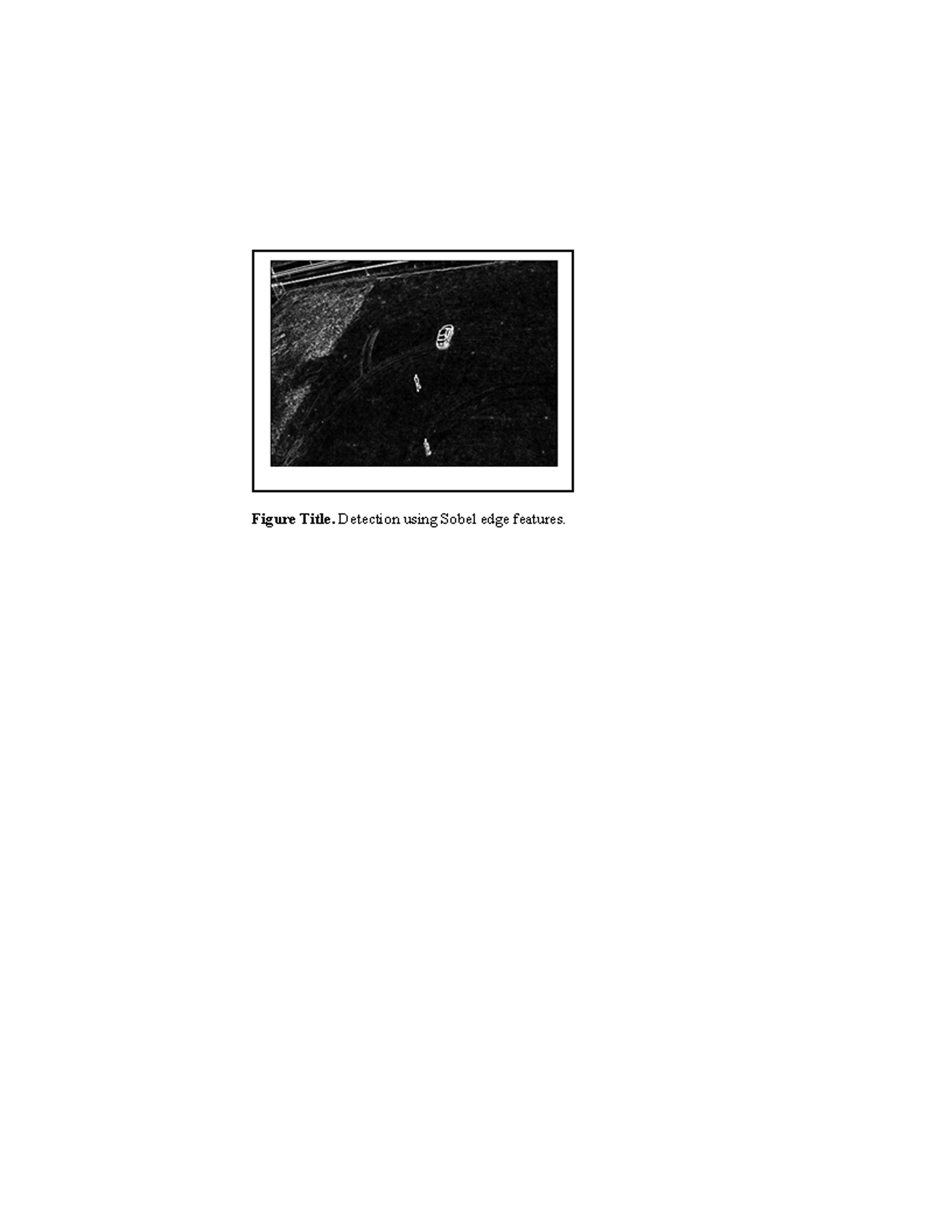

Supplement: S13 Fig — (TIF) [file pone.0126212.s013.tif]

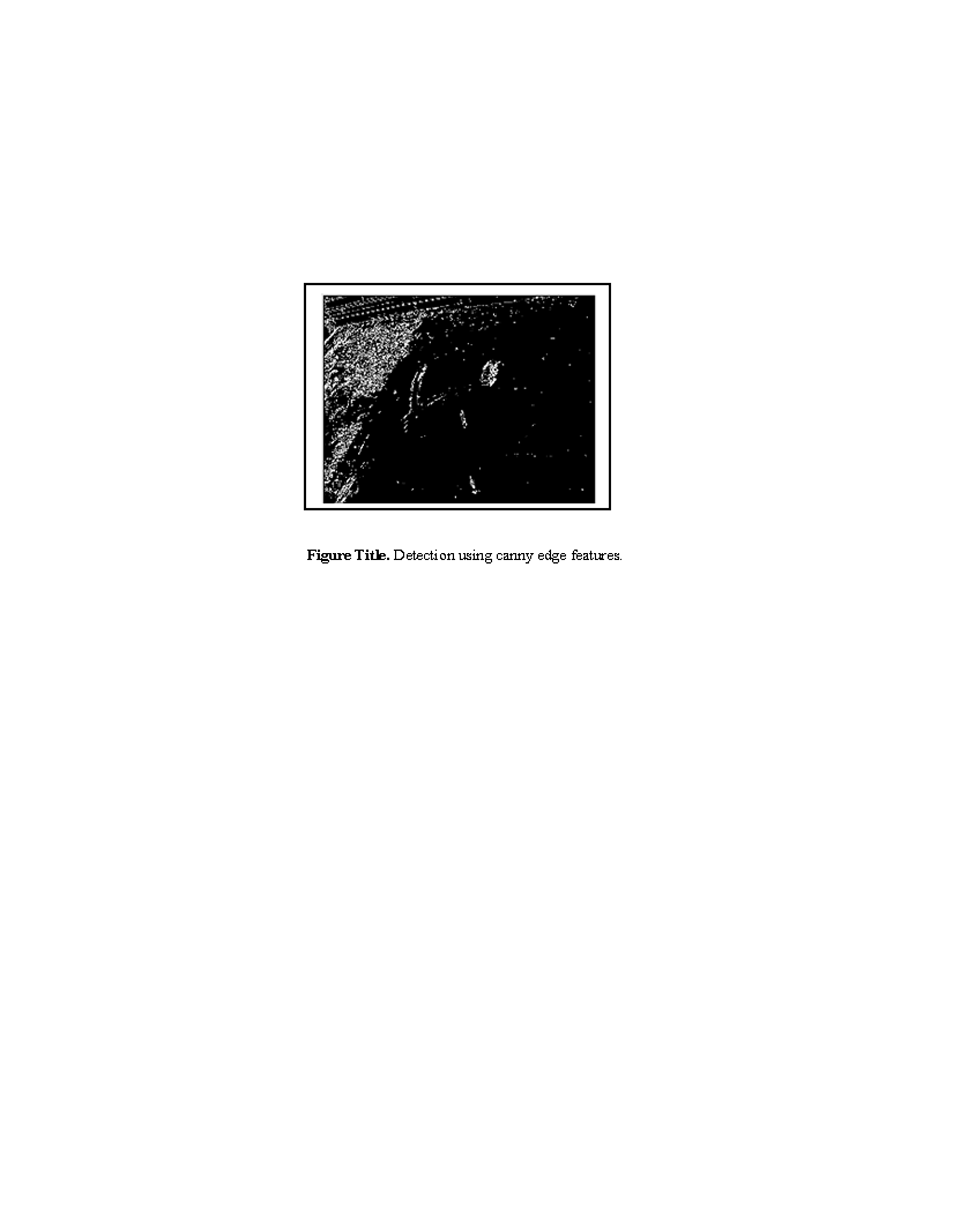

Supplement: S14 Fig — (TIF) [file pone.0126212.s014.tif]

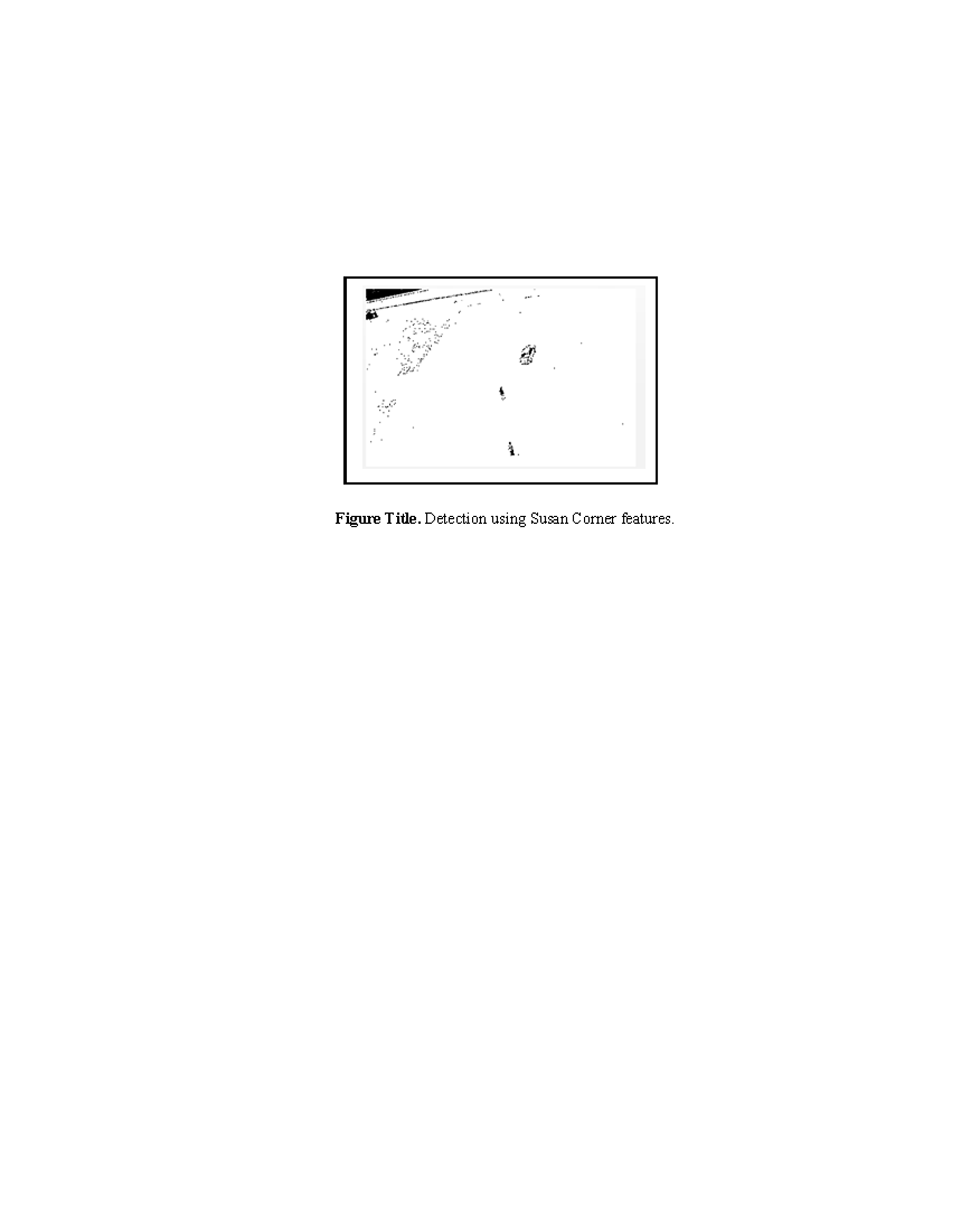

Supplement: S15 Fig — (TIF) [file pone.0126212.s015.tif]

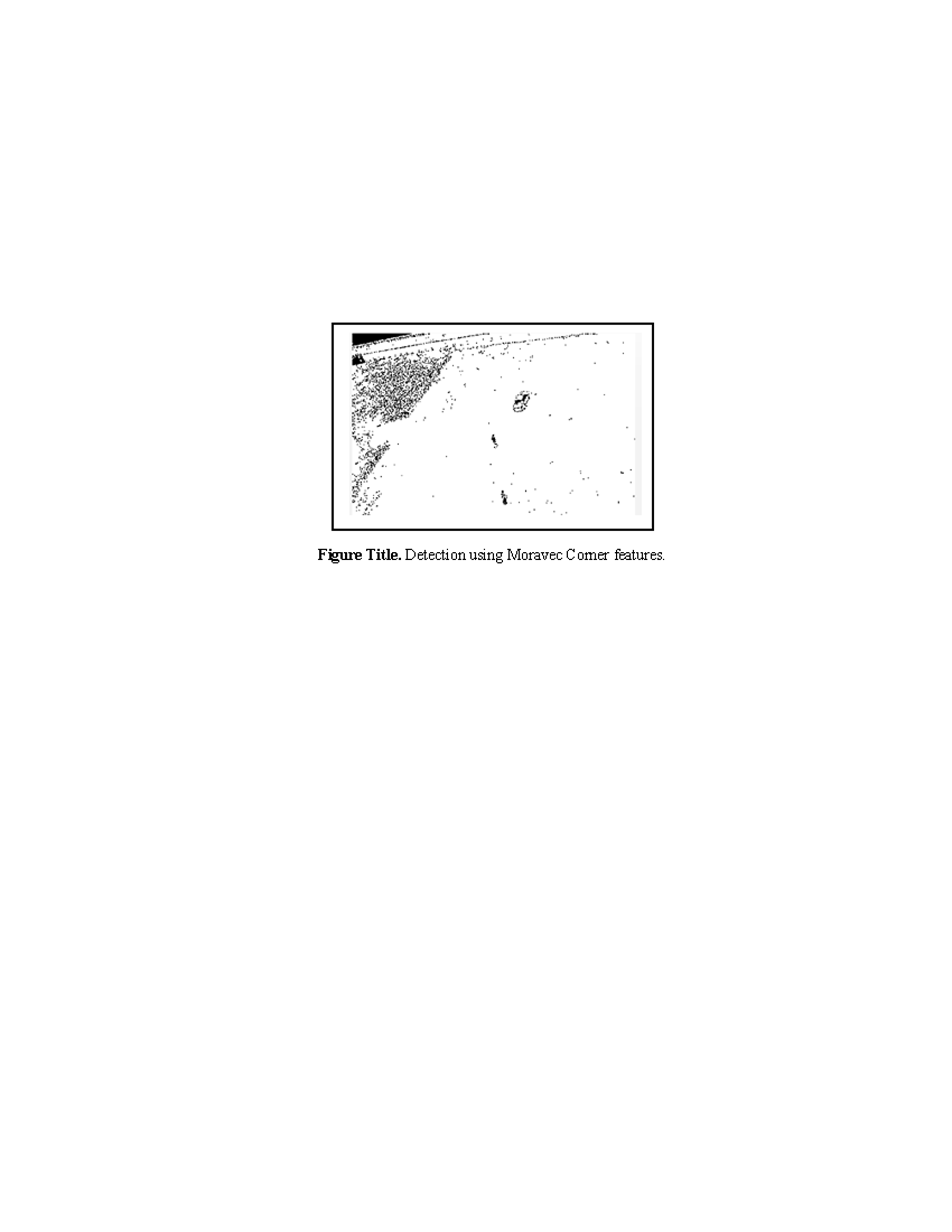

Supplement: S16 Fig — (TIF) [file pone.0126212.s016.tif]

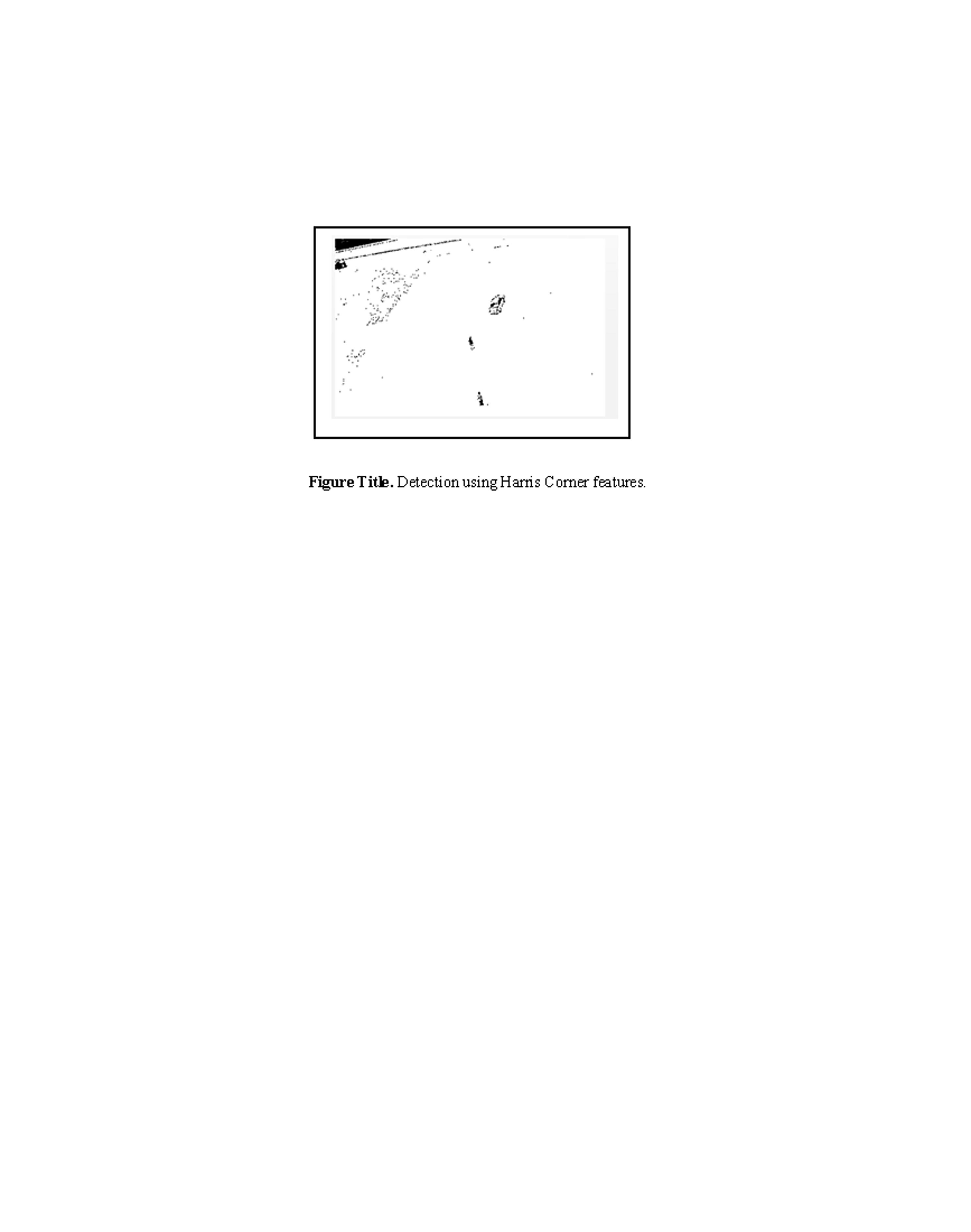

Supplement: S17 Fig — (TIF) [file pone.0126212.s017.tif]

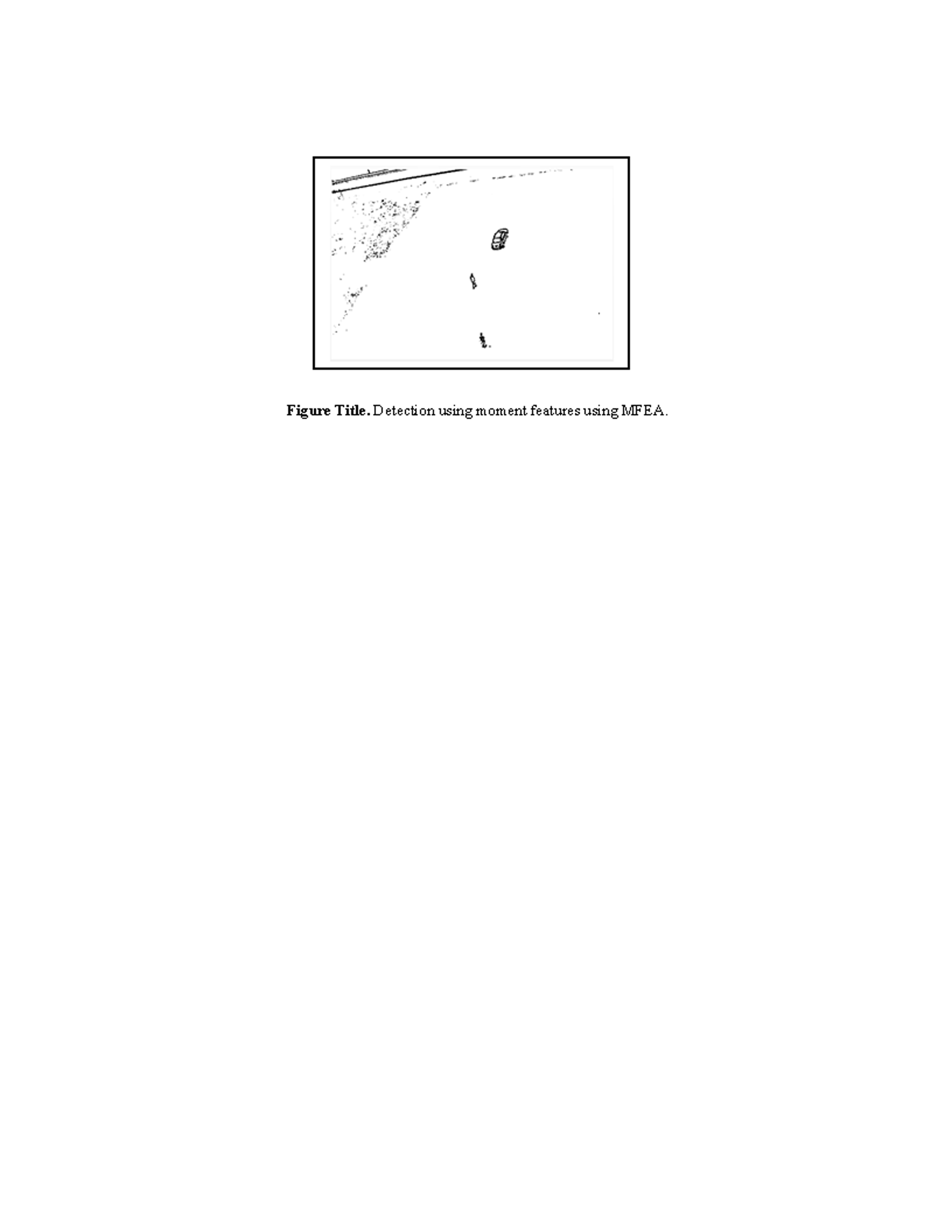

Supplement: S18 Fig — (TIF) [file pone.0126212.s018.tif]

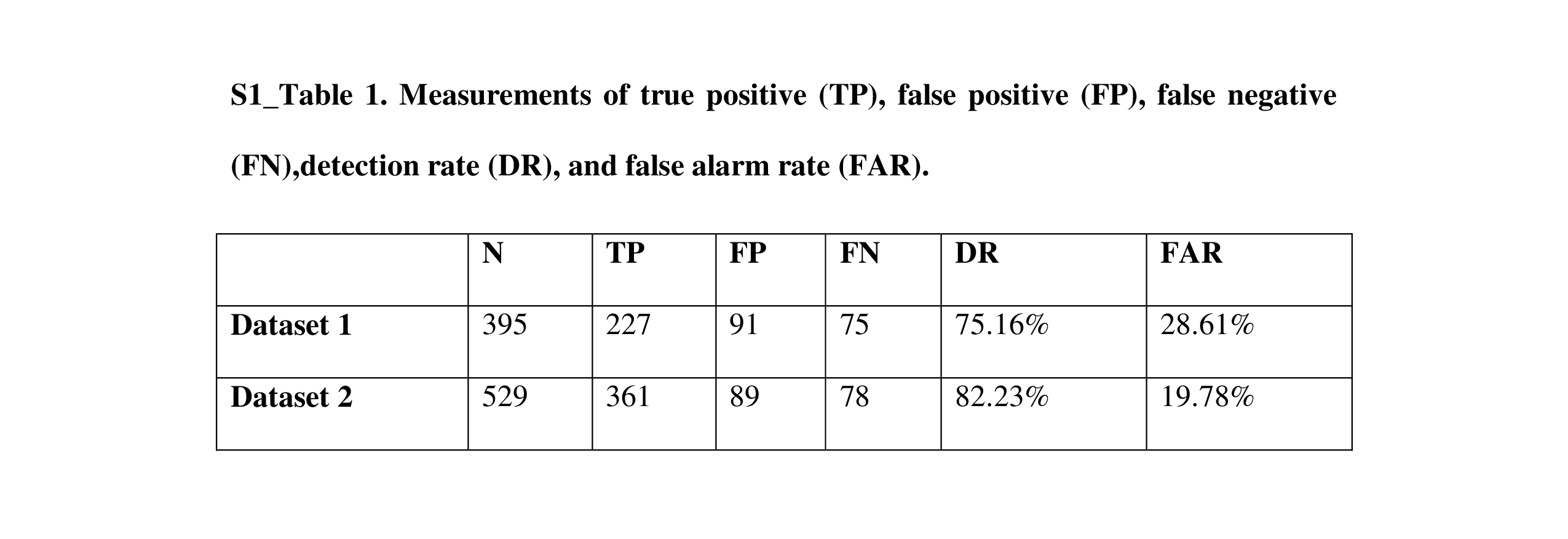

Supplement: S1 Table — (TIF) [file pone.0126212.s021.tif]
